# Protocol for the recombinant expression and purification of the LSAM domain of human legumain in *E. coli*

**DOI:** 10.1016/j.xpro.2025.103991

**Published:** 2025-07-28

**Authors:** Sven O. Dahms, Alexander C. Wieland, Hans Brandstetter, Elfriede Dall

**Affiliations:** 1 University of Salzburg, Department of Biosciences and Medical Biology, Hellbrunner Strasse 34, 5020 Salzburg, Austria; 2 Center of Tumor Biology and Immunology, University of Salzburg, 5020 Salzburg, Austria

**Keywords:** Protein Biochemistry, Protein expression and purification, Biotechnology and bioengineering

## Abstract

Expressing disulfide-rich proteins in *E. coli* is challenging due to incorrect bond formation. Here, we present a protocol for expressing the PC1pro-LSAM fusion protein in *E. coli* using the PC1 prodomain as a fusion tag and the legumain stabilization and activity modulation (LSAM) domain as a proof-of-concept target that was purified. We describe steps for characterizing the protein’s structural integrity through multiple biochemical and biophysical parameters like molecular weight, melting temperature, and secondary structure content. This protocol enables efficient expression of disulfide-containing proteins previously incompatible with bacterial systems.

## Highlights

• PC1 prodomain drives expression in *E. coli* to non-classical inclusion bodies
• PC1-pro fusion tag facilitates protein refolding from non-classical inclusion bodies
• Protocol for the preparation of functional, disulfide-linked LSAM domain
• Instructions for the expression of disulfide-bonded proteins in *E. coli*

## Abstract (editor highlights)

Publisher’s note: Undertaking any experimental protocol requires adherence to local institutional guidelines for laboratory safety and ethics.

## Graphical abstract

**Figure:**
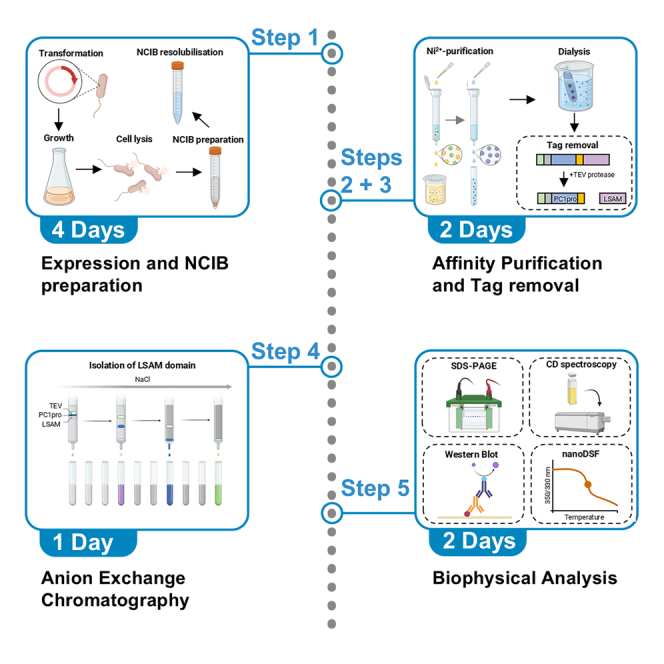

## Before you begin

**Timing: 1–2 days**

Within the endolysosomal system, the cysteine protease legumain is an important regulator in antigen processing.^1^^,^^2^^,^^3^ Synthesized as an inactive proenzyme, prolegumain is composed of a catalytic domain, an activation peptide and the C-terminal legumain stabilization and activity modulation (LSAM) domain.^4^^,^^5^^,^^6^ The LSAM domain is an important regulatory part of prolegumain, which gets released during the auto-catalytic activation. The LSAM domain contains two disulfide bonds, which made its expression particularly challenging, especially in prokaryotic systems. The construct outlined in this protocol successfully produced large amounts of pure, correctly folded protein via non-classical inclusion bodies.^7^ This protein can be used for *in vitro* and *in vivo* studies of the LSAM domain’s function, for raising antibodies against specific legumain domains and for conducting protein-protein interaction studies. The prodomains of the subtilisin-like proprotein convertases act as intermolecular chaperones and are responsible for folding and activity regulation.^8^ Prohormone Convertase 1 (PC1), a member of this family, contains an N-terminal prodomain that maintains the enzyme in an inactive, proenzyme state. Our method utilizes the PC1 (Prohormone Convertase 1, PCSK1) prodomain (PC1pro) as a fusion tag to direct expression into non-classical inclusion bodies. This fusion tag may also be effective for other proteins that are challenging to express in the *E. coli* expression systems. Additionally, the protocol may be applicable to LSAM domains from prolegumains of other organisms, such as mouse or *Arabidopsis thaliana*, and structurally homologous death domain family members.

Through the development of the herein presented protocol, we found that the design of the expression construct plays a crucial role in successful protein production. Although an N-terminally extended construct was successfully expressed in *E. coli* cells, we were unable to obtain properly folded protein. In contrast, by using the minimal construct described in this protocol we succeeded in the production of large amounts of correctly folded protein.

1. For a protein expression, prepare 2 L of TB-medium (8 × 250 mL in 2.5 L shaker flasks) in advance.
  a. Additionally, prepare at least 1 × 25 mL LB-medium in a 250 mL flask for an inoculation culture.
  b. Autoclave both and allow to cool down to room temperature (RT) before inoculation with *E. coli.*
2. Once your plasmid sequence is confirmed, transform chemocompetent BL21 (DE3) *E. coli* cells with the target plasmid using a standard heat-shock protocol to start an inoculation culture.
  a. Thaw one aliquot of chemocompetent BL21 (DE3) *E. coli* cells containing 80 μL cells on ice.
  b. Add 20 μL of 5× KCM solution.
  c. Add 100 ng of plasmid DNA to the cells.
  d. Incubate on ice for exactly 10 min.
  e. Incubate reaction mix at 42°C for exactly 30 s.
  f. Add 500 μL of LB medium to the reaction mix and immediately put at 37°C for regeneration.
  g. Regenerate cells for 1 h at 37°C in a shaker (800 rpm).
  h. After regeneration, transfer the cells into the prepared 25 mL LB-medium (1:50) with selection (e.g., 30 μg/mL Kanamycin).
  i. Grow the cells overnight (16 h) in a shaker (37°C, 200 rpm).

***Note:*** This is a protocol for polyclonal expression in *E.coli*. Monoclonal expression can be set up as well but needs more time.

***Note:*** 2×YT or LB medium may be used as well instead of TB medium. However, we expect that LB medium will result in a lower final cell density and consequently a lower overall protein yield.

3. Prepare all buffers described in materials and equipment section in advance and store them at the recommended temperatures.

***Note:*** Buffers that contain urea should always be prepared freshly and kept at 4°C to minimize carbamylation of proteins.

## Key resources table

**Table:** 

REAGENT or RESOURCE	SOURCE	IDENTIFIER
**Antibodies**		

Anti-prolegumain antiserum	BioGenes	Custom made
Secondary anti-rabbit antibody	Cell Signaling Technology Europe B.V.	7074P2

**Bacterial and virus strains**		

XL2-Blue *E. coli*	VWR	Cat#MSPP200150
BL21 (DE3) *E. coli*	Merck Millipore	Cat#69450

**Chemicals, peptides, and recombinant proteins**		

LB medium	Roth	Art.-Nr. X964.1
IPTG	Formedium	IPTG100
TB medium	Roth	Art.-Nr. HP61.1
Glycerol	AppliChem	A3739,1000
Lysozyme	Sigma-Aldrich	62971-50G-F
HEPES	AppliChem	A1069.1000
NaCl	AppliChem	A1149.5000
Tris base	AppliChem	A1086.1000
KCl	Merck	1.04936.0500
CaCl_2_	Merck	1.02382.0500
MgCl_2_	Merck	1.05833.1000
Urea	AppliChem	146392.1211
Imidazole	Merck	1.04716.1000
Glycine	Merck	1.04201.1000
Methanol	Merck	1.06009.1000
Tween 20	Sigma-Aldrich	8.22184.0500
Gloria non-fatty dry milk powder	Nestlé	012025578
Kanamycin	AppliChem	A1493.0050
DNase 1	AppliChem	A3778.0100
Ni-NTA material	QIAGEN	1018142
Source 15Q high-performance anion exchange column material	GE Healthcare	17-0947-20
TEV protease	Self-made	HIS-Tagged

**Critical commercial assays**		

GeneJET plasmid miniprep kit	Thermo Fisher Scientific	Cat#K0502

**Other**		

Spectra/Por dialysis membrane (MWCO: 6–8 kDa)	Spectrum Laboratories Inc.	132 650
Amicon Ultra centrifugal filter (MWCO: 3 kDa)	Merck	UFC 900 324
PVDF membrane	Merck	ISEQ85R
Liquid chromatography columns	Merck	C4669-5EA
PC1pro-LSAM expression plasmid	Self-made; deposited to Addgene	240215

## Materials and equipment

**Table:** TB medium

Reagent	Final concentration	Amount for 1 L
TB-medium	N/A	47.6 g
Glycerol	0.4% (v/v)	4 mL

***Note:*** Store at 22°C for up to 6 months.

**CRITICAL:** Add Glycerol before autoclaving.

**CRITICAL:** Autoclave after preparation (121°C, 20 min).

**Table:** LB medium

Reagent	Final concentration	Amount for 1 L
LB-medium	N/A	20 g

***Note:*** Store at 22°C for up to 6 months.

**CRITICAL:** Autoclave after preparation (121°C, 20 min).

**Table:** 5×-KCM

Reagent	Final concentration	Amount for 100 mL
KCl	0.5 M	3.72 g
CaCl_2_	0.15 M	1.66 g
MgCl_2_	0.25 M	2.38 g

***Note:*** Store at −20°C for up to 6 months.

**CRITICAL:** Sterile-filter after preparation.

**Table:** Ni-buffer A

Reagent	Final concentration	Amount for 1 L
Tris/HCl	100 mM	12.11 g
NaCl	500 mM	29.22 g

***Note:*** Store at 4°C for up to 1 month.

**CRITICAL:** Adjust pH to 8.0 at RT with HCl or NaOH.

**Table:** Ni-buffer B

Reagent	Final concentration	Amount for 1 L
Tris/HCl	100 mM	12.11 g
NaCl	500 mM	29.22 g
Imidazole	500 mM	34.03 g

***Note:*** Store at 4°C for up to 1 month.

**CRITICAL:** Adjust pH to 8.0 at 22°C with HCl or NaOH.

**Table:** Urea buffer

Reagent	Final concentration	Amount for 1 L
Urea	8 M	480.48 g
Tris/HCl	50 mM	6.05 g

***Note:*** Store at 4°C for up to 1 month.

**CRITICAL:** Adjust pH to 8.0 at 22°C with HCl or NaOH.

**Table:** IEX-buffer A

Reagent	Final concentration	Amount for 1 L
Tris/HCl	20 mM	2.42 g

***Note:*** Store at 22°C for up to 1 week.

**CRITICAL:** Adjust pH to 8.0 at 22°C with HCl or NaOH.

**Table:** IEX-buffer B

Reagent	Final concentration	Amount for 1 L
Tris/HCl	20 mM	2.42 g
NaCl	1 M	58.44 g

***Note:*** Store at 22°C for up to 1 week.

**CRITICAL:** Adjust pH to 8.0 at 22°C with HCl or NaOH.

**Table:** Dialysis buffer

Reagent	Final concentration	Amount for 2 L
Tris/HCl	20 mM	4.84 g
NaCl	50 mM	5.84 g

***Note:*** Store at 4°C for up to 1 week.

**CRITICAL:** Adjust pH to 8.0 at 22°C with HCl or NaOH.

**Table:** Storage buffer

Reagent	Final concentration	Amount for 1 L
Tris/HCl	20 mM	2.42 g
NaCl	85 mM	4.96 g

***Note:*** Store at 4°C for up to 1 week.

**CRITICAL:** Adjust pH to 8.0 at 22°C with HCl or NaOH.

**Table:** Assay buffer

Reagent	Final concentration	Amount for 1 L
Tris/HCl	20 mM	2.42 g
NaCl	100 mM	5.84 g

***Note:*** Store at 22°C for up to 1 week.

**CRITICAL:** Adjust pH to 8.0 at 22°C with HCl or NaOH.

**Table:** Transfer buffer

Reagent	Final concentration	Amount for 100 mL
Tris/HCl	25 mM	0.60 g
Glycine	192 mM	1.44 g
Methanol	10% (v/v)	10 mL

***Note:*** Store at 22°C, freshly prepared (only the amount needed)

**Table:** 10× TBS

Reagent	Final concentration	Amount for 1 L
Tris/HCl	200 mM	24.20 g
NaCl	1.5 M	87.66 g

***Note:*** Store at 22°C for up to 1 month.

**CRITICAL:** Adjust pH to 7.6 at 22°C with HCl or NaOH.

**Table:** 1× TBS

Reagent	Final concentration	Amount for 1 L
Tris/HCl	20 mM	2.42 g
NaCl	150 mM	8.80 g

***Note:*** Store at 22°C for up to 1 week.

**Table:** TBST

Reagent	Final concentration	Amount for 1 L
Tris/HCl	20 mM	2.42 g
NaCl	150 mM	8.80 g
Tween-20	0.1% (v/v)	1 mL

***Note:*** Store at 22°C for up to 1 week.

**Table:** Blocking solution

Reagent	Final concentration	Amount for 50 mL
Tris/HCl	20 mM	0.12 g
NaCl	150 mM	0.44 g
Non-fatty dry milk	5% (w/v)	2.5 g

***Note:*** Store at −20°C for up to 6 months.

## Step-by-step method details

### Production of recombinant LSAM domain in non-classical inclusion bodies

**Timing: 4 days**

This section describes the production of the LSAM domain as non-classical inclusion bodies. . In contrast to classical inclusion bodies, non-classical inclusion bodies are protein aggregates that contain folded protein, are easily soluble and allow the extraction of target proteins under non-denaturing conditions.^9^ It includes three major steps: Plasmid preparation (1 day), protein expression (1 day) and purification of non-classical inclusion bodies (2 days).

**Figure 1:**
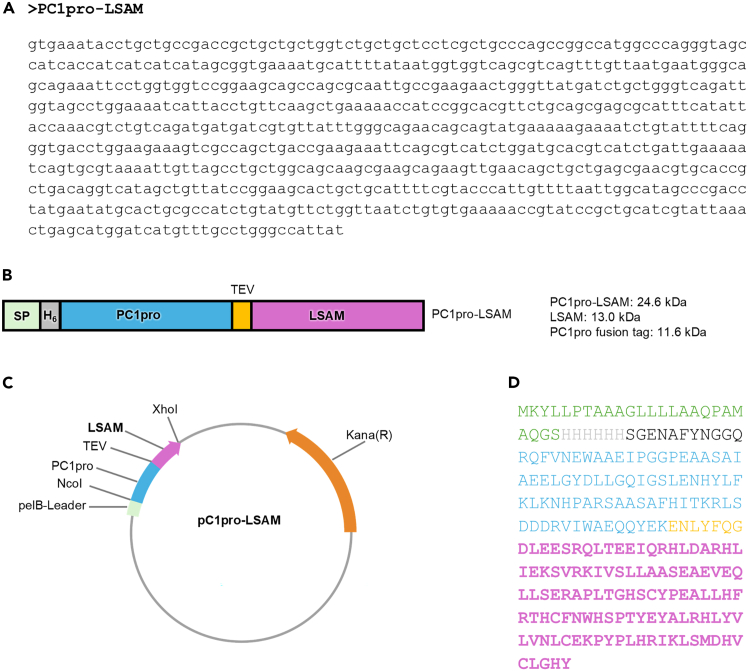
Scheme of the PC1pro-LSAM fusion construct (A) Sequence of the PC1pro-LSAM expression construct. (B) The expression construct comprised of an N-terminal pelB signal peptide for periplasmic expression (SP), an N-terminal His_6_-tag, followed by the PC1-pro fusion tag, and the TEV-recognition sequence linked to the N-terminal end of the LSAM domain. (C) Vector map of the PC1pro-LSAM fusion construct. (D) Amino acid sequence of the resulting protein.

**Figure 2:**
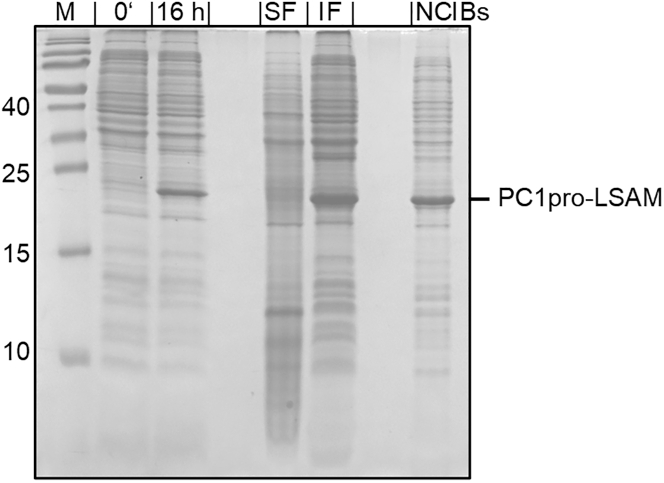
Expression and NCIB preparation of the PC1pro-LSAM fusion protein Expression of PC1pro-LSAM was carried out in *E. coli* BL21(DE3) cells, overnight (16 h) at 24°C after induction with IPTG. Cells are lysed by sonication and the PC1Por-LSAM fusion protein retains in the insoluble fraction (IF) containing the NCIBs. The pellet is washed and finally solubilized in buffer containing 3 M urea (NCIBs). SF: soluble fraction after sonication.

1. Plasmid Preparation.
  ***Note:*** This section describes the preparation of plasmids containing the expression construct of the LSAM domain. The plasmid preparation contained 3 sequential steps: (i) synthesis of the expression construct by a provider, (ii) subcloning (by a provider), (iii) preparation of plasmid DNA.
  a. Synthesis of the expression construct.
    i. Order the DNA of the PC1pro-LSAM expression construct from a DNA synthesizing company of your choice.
    ii. Include 5′ NcoI and 3′ XhoI restriction enzyme cleavage sites.
      ***Note:*** The LSAM sequence used comprised of amino acids Asp324-Tyr433 (UniProt: Q99538-1).^5^
      ***Note:*** The PC1pro fusion tag comprised of amino acids 28 – 110 (UniProt: P29120) carrying the mutations H72L, R77A, R80A and R81A.
      ***Note:*** For this protocol the sequence for PC1pro-LSAM was codon optimized for *E. coli* expression. The FASTA sequence can be found in Figure 1A.
      ***Note:*** If the construct is prepared in the pET28B(+) expression vector, 5′ NcoI and 3′ XhoI restriction enzyme cleavage sites should be used for inserting the expression construct. If another expression vector is used, different enzymes may be used accordingly.
  b. Subcloning of the expression construct.
    i. Subclone the DNA into the expression vector (e.g., pET-28b(+), Novagen).
    ii. Use your preferred cut-and-paste cloning protocol or use a subcloning service by a provider.
  c. Preparation of plasmid DNA.
    i. Transfer plasmid DNA into *E. coli* (e.g., XL2-Blue) by electroporation.
    ii. Regenerate transformation culture in 500 μL pre-warmed (37°C) LB-Medium on a shaker incubator for 1 h.
    iii. Transfer 20 μL of regenerated cells into 4 mL LB-medium supplemented with 30 μg/mL Kanamycin.
    iv. Grow at 37°C and 200 rpm overnight (16 h) in a shaker incubator.
    v. Extract plasmid DNA with GeneJet MiniPrep Kit (Thermo Fisher) according to manufacturer. Other products should work as well.
      ***Note:*** For the procedure described in this protocol we subcloned our expression construct into the pET-28b(+) expression vector. However, other vectors may work as well, as long as the expression construct (PC1pro fusion tag linked to target protein) remains the same.
      ***Note:*** Instead of electrocompetent XL2-Blue cells, other electrocompetent or chemocompetent E. coli strains will similarly work.
      ***Note:*** The expression construct described in this protocol was deposited to Addgene: 240215.
2. Expression of PC1pro-LSAM fusion protein in non-classical inclusion bodies.
  ***Note:*** This section describes the expression of the PC1pro-LSAM fusion protein as non-classical inclusion bodies in bacterial cultures and the follow up purification of the non-classical inclusion bodies. It contains 3 sequential steps: Start of inoculation culture, expression, and harvest (Figure 2).
  ***Note:*** The PC1pro fusion tag drives expression into non-classical inclusion bodies.^7^
  a. Inoculation culture.
    ***Note:*** After screening different expression strains, BL21 (DE3) *E. coli* gave the best expression results in pre-experiments. However, other BL21 (DE3) derivatives may work as well.
    i. Plasmids, which were confirmed by sequencing to have the PC1pro-LSAM gene, are transferred into BL21 (DE3) *E. coli cells* using a heat shock protocol (see section ‘before you begin’, step 2).
    ii. After regeneration transfer the bacterial cells into 25 mL LB-medium (containing 30 μg/mL Kanamycin, corresponding to the resistance provided by the plasmid).
    iii. Grow the bacterial cells in a 37°C incubator overnight (16 h) at 200 rpm shaking.
  b. Expression culture and expression start.
    i. Inoculate 8 × 250 mL TB-medium (in 2.5 l baffled flasks, contains 7.5 μg/mL Kanamycin) with 2.5 mL each of inoculation culture.
    ii. Grow in an incubator at 37°C and 200 rpm shaking to an OD600 of 0.5 (1.5–2 h).
    iii. Transfer cultures to 24°C (pre-cooled incubator, 200 rpm shaking).
    iv. Grow cultures until OD600 ≥ 10 (∼6 h).
    v. Induce expression by adding 1 mM IPTG.
    vi. Grow cultures overnight (16 h) and measure OD600 (expected OD600 = 12–13).
      ***Note:*** We expect that expression may also be carried out at temperatures <24°C.
  c. Harvest.
    i. Harvest cultures by centrifugation at 4°C (15 min, 4000 g).
    ii. Discard medium and transfer cells of 500 mL culture to 50 mL tubes (pool 2 pellets into one 50 mL tube).
    iii. Store the pooled pellets at −20°C.
      ***Note:*** For long-term storage of the pooled pellets, we recommend storing them at −80°C.

### Isolation of non-classical inclusion bodies

This section describes the isolation of the PC1pro-LSAM fusion protein from NCIBs from the harvested bacterial pellets. It contains 3 sequential steps: Cell lysis, Wash and NCIB resolubilization (Figure 2).

3. Cell lysis.
  a. Thaw bacteria pellets at room temperature.
  b. Prepare 5 mL of 30 mg/mL lysozyme in Ni-buffer A at room temperature.
  c. Resuspend bacteria in 30 mL Ni-buffer A per 500 mL of *E.coli* culture at room temperature.
  d. Add 1 mM CaCl_2_ (30 μL of 1 M CaCl_2_ stock solution), 2.5 mM MgCl_2_ (75 μL of 1 M MgCl_2_ stock solution) & spatula tip of lyophilized DNase (approx. 5000 U/mg) per tube.
  e. Add 1 mL lysozyme solution per tube.
  f. Rotate for 1 h at room temperature.
  g. Cool tubes 10 min on ice before sonication.
  h. Sonicate on ice at 25% power, 20% cycle (200 ms pulse, 800 msec brake), for 3 min.
  i. Repeat this step 4 times.
  j. Mix bacteria suspensions between sonication runs.
  k. Centrifuge at 17500 g and 4°C for 10 min and Remove the supernatant.
  l. Centrifuge at 17500 g and 4°C for 2 min and,
  m. Remove residual supernatant.
4. Washing of NCIBs.
  a. Resuspend pellet in 30 mL Ni-buffer A at room temperature.
  b. Centrifuge at 17500 g for 10 min at 4°C.
  c. Remove the supernatant.
  d. Centrifuge at 17500 g and 4°C for 2 min.
  e. Remove residual supernatant.
  f. Repeat wash step once.
5. NCIB resolubilization.
  a. Resuspend NCIBs in 30 mL premixed and ice cold solubilization buffer (62.5% Ni-buffer A and 37.5% Urea buffer), final Urea concentration is 3 M.
  b. Rotate solubilization mix at 4°C overnight (16 h).
  c. Centrifuge at 17500 g and 4°C for 10 min.
  d. Collect supernatant with solubilized fusion protein in a 50 mL falcon tube.
  e. Immediately use it for Ni^2+^-purification.

### Ni^2+^-affinity purification of the PC1pro-LSAM fusion protein

**Timing: 1 day**

This section describes the purification of the PC1pro-LSAM fusion protein via Ni^2+^-affinity purification. It contains 3 major steps: Equilibration of the Ni-NTA material, Washing and Elution.

**Figure 3:**
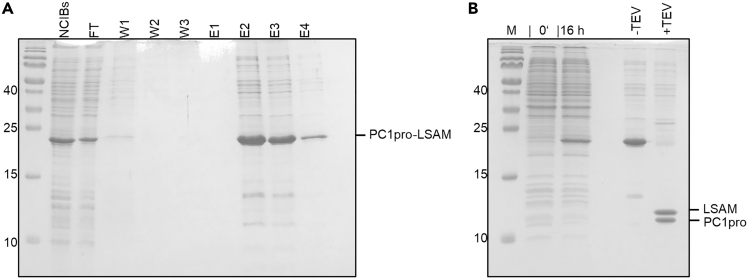
Ni^2+^-purification and subsequent TEV digestion of the PC1pro-LSAM fusion protein (A) The PC1pro-LSAM fusion protein binds to the Ni^2+^-resin via its N-terminal His_6_-tag. FT: Flow through, W1-W3: Wash 1 – Wash 3; E1 – E4: Elution 1 – Elution 4. (B) After elution and dialysis, TEV protease was added to the sample to release the isolated LSAM domain from the PC1pro fusion tag (0’: Cells before induction; 16 h: cells 16 h after induction).

6. Equilibration of the Ni-NTA material.
  ***Note:*** This section describes the equilibration of the Ni-NTA column material prior to protein purification. It contains three sequential steps: Preparation of buffers, equilibration of Ni-NTA material, loading of resolubilized NCIBs.
  a. Preparation of buffers.
    i. Prepare 200 mL of Wash buffer 1 by addition of 2% (4 mL) Ni-buffer B to Ni-buffer A (196 mL, resulting in 10 mM imidazole final concentration).
    ii. Prepare 50 mL of Wash buffer 2 by addition of 4% Ni-buffer B to Ni-buffer A (20 mM imidazole final concentration).
    iii. Prepare 30 mL elution buffer by addition of 50% Ni-buffer B to Ni-buffer A (250 mM imidazole final concentration).
  b. Equilibration of Ni-NTA material.
    i. Equilibrate 5 mL Ni-NTA material (10 mL of 50% suspension) with 2 × 25 mL Wash buffer 1 by gravity flow.
    ii. Add 0.6 mL Ni-buffer B to solubilized protein (to obtain a concentration of 10 mM imidazole in the sample) and mix well.
  c. Loading of resolubilized NCIBs.
    i. Resuspend Ni-NTA material in 10 mL Wash buffer 1 and transfer to solubilized protein.
    ii. Resuspend remaining Ni-NTA material (sticking to the wall of the gravity flow column) in another 5 mL Wash buffer 1 and transfer to solubilized protein.
    iii. Incubate at 4°C for 30 min under rotation.
7. Washing and elution of the Ni-NTA material.
  ***Note:*** This section describes the washing and the elution of the Ni-NTA material after the PC1pro-LSAM fusion protein was loaded and bound to the beads. It contains three major steps: Transfer of beads to the column, washing and elution.
  a. Transfer of beads to the column.
    i. Harvest the Ni^2+^-beads back into the column, empty by gravity flow and collect flow through.
    ii. Use a pipette boy to increase the flow rate as much as possible.
    iii. Some beads will stick to the inside of the glass bottle. Wash them out by adding 25 mL Wash buffer 1, shake well and pour the wash into the column.
    iv. Empty the column by gravity flow.
    v. Repeat this step twice.
  b. Washing.
    i. Add 50 mL Wash buffer 1 to the beads and empty the column by gravity flow.
    ii. Add 50 mL Wash buffer 2 to the beads and empty the column by gravity flow.
  c. Elution.
    i. Add 3 mL elution buffer to the beads and let the buffer slowly migrate through the column by gravity flow.
    ii. Add 10 mL elution buffer to the beads and let the buffer slowly migrate through the column by gravity flow.
    iii. Add 5 mL elution buffer and let the buffer slowly migrate through the column by gravity flow.
    iv. Add 5 mL elution buffer and let the buffer slowly migrate through the column by gravity flow.
    v. Elutions can be frozen at −20°C until further use.
      ***Note:*** Inspect success of Ni^2+^-purification by loading samples on an SDS-PAGE gel (Figure 3A). Determine amount of sample to be loaded on the gel by measuring the UV_280_ signal (extinction coefficient: 1.343 AU/mg/mL). Determine the volume that is required for 2.5 μg of protein of the fraction with the highest protein centration. Load this volume of all elution fractions to the gel. Whether target proteins other than PC1pro-LSAM can be stored by freezing and by which freezing protocol (e.g., drop freezing in lN2) must be tested and established individually for each target protein.

### Tag removal of the recombinant LSAM domain

**Timing: 2 days**

This section describes the removal of the PC1pro fusion tag from the LSAM domain. It contains 2 major steps: dialysis (1 day) and digestion with TEV protease (1 day).

8. Dialysis.
  a. Pool elutions containing PC1pro-LSAM fusion protein.
  b. Determine protein concentration by measuring the UV_280_ signal.
  c. Transfer pooled elutions to dialysis tubing (Molecular weight cutoff 6 – 8 kDa) preequilibrated in dialysis buffer.
  d. Transfer dialysis tubing to at least 100× of the volume of the pooled elutions and dialyze 1 h at room temperature (stir gently). E.g. for 10 mL sample use 1 l dialysis buffer.
  e. Transfer dialysis tubing to fresh dialysis buffer and dialyze for another 3 h at room temperature (stir gently).

***Note:*** Dialysis can also be done at 4°C instead of room temperature. In that case dialyze first 3 h at 4°C, then do a buffer exchange and do the second dialysis step overnight (16 h) at 4°C.

9. TEV digestion.
  a. Take out protein sample from dialysis tubing after the second dialysis step and transfer to a Falcon tube.
  b. Measure protein concentration using the Bradford assay or by measuring the UV_280_ signal.
  c. Add TEV-protease in a 1:20 ratio (m/m).
  d. Digest overnight (16 h) at room temperature.

***Note:*** It is recommended to analyze the success of the TEV digestion by loading samples on an SDS-PAGE gel. Load an undigested sample as a negative control (Figure 3B).

***Note:*** In our lab, we use a home-made TEV protease harboring S219N-N68D-I77V-T17S mutations that increase its solubility.^10^ However, a commercially available TEV protease will similarly work. In that case, however, it is recommended to test different ratios of TEV protease : protein sample before setting up the final digestion reaction.

### Isolation of pure LSAM domain

**Timing: 1 day**

This section describes the separation of the LSAM from the PC1pro-fusion tag and the TEV protease via Anion-exchange chromatography. It contains 2 major steps: Anion-exchange chromatography and concentration of purified LSAM domain.

10. Anion-exchange chromatography.
  ***Note:*** This section describes the separation of the isolated LSAM domain from free PC1pro-fusion tag, uncleaved PC1pro-LSAM fusion protein, and TEV protease via anion-exchange chromatography (IEX). It contains three sequential steps: Loading, Washing and Elution (Figure 4).
  a. Loading.
    i. Equilibrate a Source 15Q high performance anion exchange column (column volume 2 mL) at a flow rate of 0.5 mL/min with 5% IEX-buffer A (50 mM NaCl) until a constant baseline is reached.
    ii. Apply protein to the column using a suitable loop (e.g. 10 mL loop).
    iii. Fractionate flow through (FT) in 2 mL fractions.
      ***Note:*** Do not load more than 30 mg overall protein to the column. If you got more protein after your TEV digestion, do several rounds of anion-exchange chromatography or use a bigger Source 15Q column.
  b. Washing.
    i. Wash with 5% IEX-buffer A (50 mM NaCl) until a constant baseline is reached.
    ii. Collect 2 mL-fractions.
  c. Elution.
    i. The first elution step is done by increasing the concentration of NaCl to 8.5% at a flow rate of 0.5 mL/min.
    ii. Collection 0.5 mL fractions.
    iii. Continue this elution step until the UV_280_ signal went down to baseline again.
    iv. The second elution step is done by increasing the concentration of NaCl to 25% at a flow rate of 0.5 mL/min.
    v. Collect 1 mL fractions.
    vi. Continue this elution step until the UV_280_ signal went down again.
    vii. The third elution step is done by setting the concentration of NaCl to 100% (100% IEX-buffer B) at a flow rate of 0.5 mL/min. This typically takes approx. 10 min.
    viii. Collect 1 mL-fractions.
    ix. Stop elution when UV_280_ signal went down to baseline again.
    x. Analyze elution fractions by SDS-PAGE.
    xi. Measure protein concentration in elution fractions and determine the volume that is required for 2.5 μg of protein of the fraction with the highest protein concentration.
    xii. Load this volume of all elution fraction to the gel.
    xiii. Pool fractions containing isolated LSAM domain.
    xiv. Samples can be frozen until further use.

**Figure 4:**
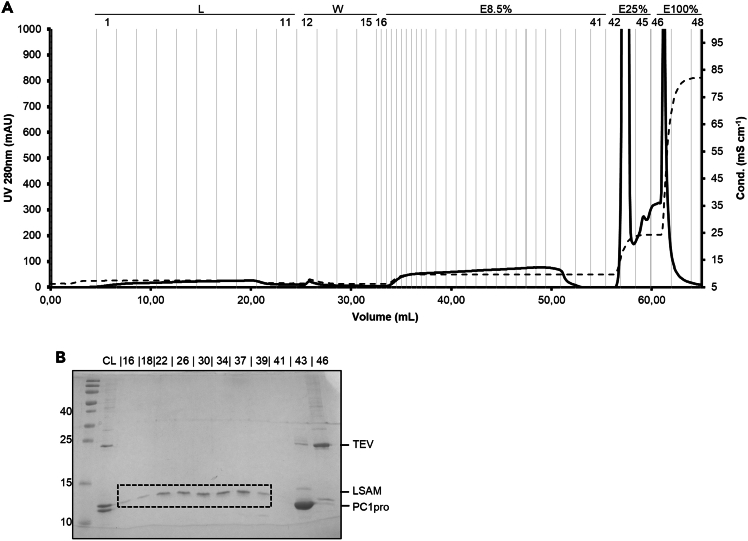
Anion-exchange chromatogram of the TEV digested reaction (A) An anion-exchange chromatography is carried out to separate the LSAM domain from the PC1pro-fusion tag and the TEV protease. Dashed line: conductivity, solid line: UV_280_ signal. (B) After digestion of the PC1pro-LSAM fusion protein, three bands are visible in the load for the anion-exchange chromatography: TEV protease, the LSAM domain and the PC1pro-fusion tag. The LSAM domain eluted in the first elution step (8.5% NaCl, dashed box), the PC1-pro fusion tag in the second elution step (25% NaCl) and the TEV protease in the final elution (100% NaCl).
11. Preparation of protein for storage.
  ***Note:*** This section describes the final concentration of the purified LSAM domain and its storage. It contains two sequential steps: Concentration of LSAM domain and storage of LSAM domain.
  a. Concentration of LSAM domain.
    i. Pool fractions of IEX chromatography run that contain free LSAM domain.
    ii. Concentrate sample using an Amicon Ultra centrifugal filter (MWCO: 3 kDa) to a final volume of approx. 1 mL @ approx. 2 mg/mL final protein concentration.
    iii. Determine protein concentration by measuring the UV_280_ signal. The final concentration should be 5 – 10 mg/mL.
    iv. Transfer concentrated LSAM domain to thin-walled PCR tubes. Put 20 μL of LSAM domain into each tube.
    v. Store protein at −20°C.

### *In vitro* characterization of the LSAM domain

**Timing: 2 days**

This section describes *the in vitro* characterization of purified LSAM domain by SDS-PAGE (4 h), CD spectroscopy (4 h), nanoDSF measurements (1 h) and a western blot (1 day).

12. Analysis of purity by SDS-PAGE.
  a. Mix 2.5 μg of LSAM protein with an appropriate amount of 2× SDS-PAGE loading buffer containing reducing agent.
  b. Mix 2.5 μg of LSAM protein with an appropriate amount of 2× SDS-PAGE loading buffer not containing reducing agent.
  c. Run an SDS-PAGE gel.

**Figure 5:**
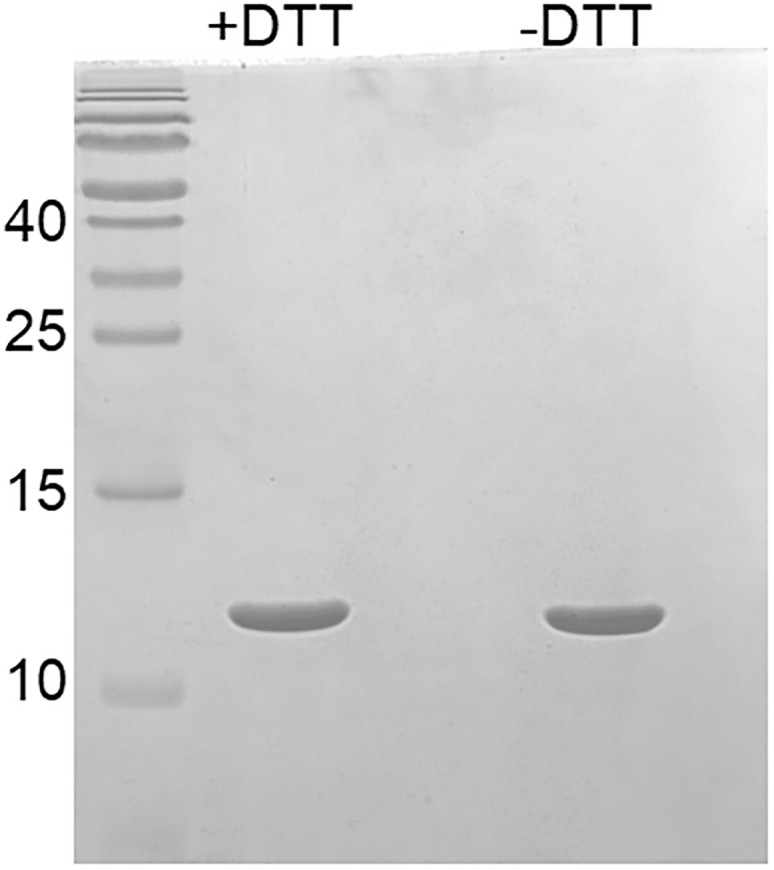
SDS-PAGE of the LSAM domain with and without reducing agent DTT When the purified LSAM domain is loaded on the SDS-PAGE gel without a reducing agent, it migrates faster than in the presence of a reducing agent. This is consistent with the presence of two disulfide bonds, which make the protein appear apparently smaller on a non-reducing SDS-PAGE gel.

***Note:*** It is recommended to use 15% polyacrylamide gels in order to have a good resolving power for small proteins (<15 kDa) and middle-sized proteins (<50 kDa). It is recommended to analyze samples with both reducing (+DTT) and non-reducing (-DTT) SDS loading buffer. Since the LSAM domain contains two disulfide bonds, the non-reducing sample should migrate slightly faster than the reducing sample (Figure 5).^5^ Incorrectly folded cysteine-containing domains tend to form intermolecular disulfide bonds, resulting in higher order oligomer bands (dimer, trimer, etc.) on a non-reducing gel.^11^

***Note:*** Samples do not need to be boiled before loading. Freshly taken samples that are not immediately loaded on a gel can be stored at 4°C.

***Note:*** The SDS PAGE samples are particularly helpful for establishing or trouble shooting the protocol. Therefore, we recommend taking samples for SDS-PAGE after all steps.

13. Analysis of proper folding by Circular Dichroism Spectroscopy.
  ***Note:*** This section describes the analysis of the secondary structure content of the purifies LSAM domain by Circular Dichroism (CD) Spectroscopy (Figure 6). Far UV CD spectra were recorded using a Chirascan Plus CD-spectrophotometer (Applied Photophysics) equipped with a Peltier temperature-controlled cuvette holder at 20°C in a QS high precision cell with 1 mm path length (Hellma Analytics). It contains two sequential steps: Sample preparation and CD-spectroscopy measurements.
  a. Sample preparation.
    i. Prepare 250 μL of LSAM sample with a concentration of 5 μM.
    ii. Use assay buffer to dilute the protein.
    iii. Transfer sample into the cuvette used for the subsequent measurements.
  b. CD-spectroscopy measurements.
    i. Before starting the measurement, the instrument is flushed with nitrogen, the spectral bandwidth is set to 1 nm and the scan time per point to 1 s.
    ii. The Peltier temperature-controlled cuvette holder is set to 20°C.
    iii. CD spectra from 200 to 260 nm are recorded.

**Figure 6:**
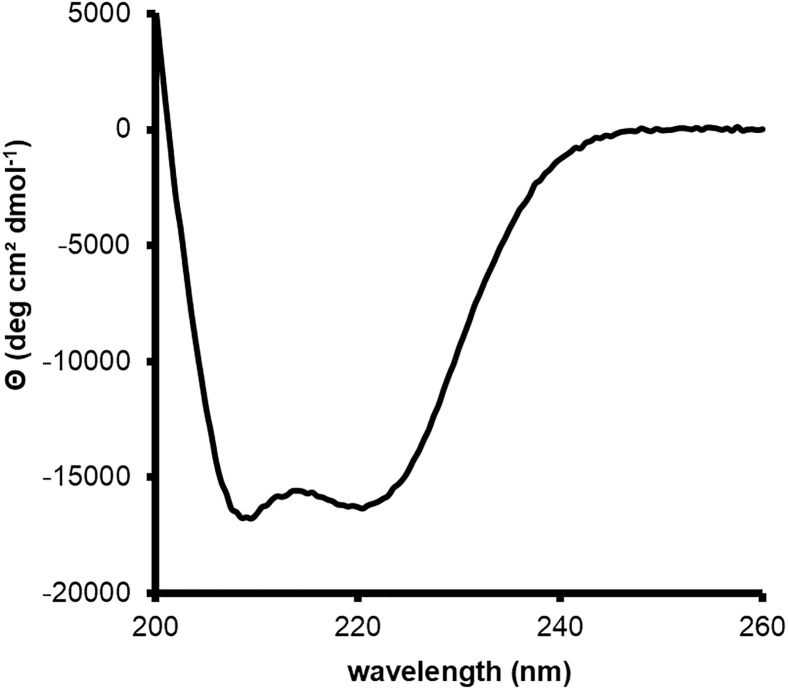
CD-spectroscopy measurement of the purified LSAM domain at pH 8.0 The spectrum is consistent with an all-helical protein with two negative peaks of similar magnitude at 208 nm and 222 nm and a positive peak at approx. 190 nm.
14. Analysis of folding by nanoDSF measurements.
  ***Note:*** This section describes the analysis of the thermal stability of the LSAM domain by nanoDSF (Differential Scanning Fluorimetry) measurements. The measurement was performed by using the Tycho NT.6 instrument (NanoTemper Technologies). Changes in intrinsic fluorescence intensity of tryptophane and tyrosine residues are measured at 330 and 350 nm upon heating the sample from 35°C to 95°C within a total measuring time of approx. 3 min. From the unfolding profile the inflection temperature (T_i_) was determined, which indicated the unfolding transition temperature.^12^^,^^13^
  a. nanoDSF measurement.
    i. Prepare 35 μL of LSAM sample with a concentration of 1 mg/mL by diluting sample in storage buffer.
    ii. Spin at 16,000 g for 30 s at RT.
    iii. Fill 3 capillaries with sample.
    iv. Record the intrinsic fluorescence at 330 nm and 350 nm, while heating the sample from 35°C to 95°C (20°C per min).
    v. The ratio of fluorescence 350/330 nm and the inflection temperature T_i_ are calculated by the Tycho NT.6 software.
    vi. Plot the normalized fluorescence ratio vs. temperature.
      ***Note:*** Spinning the samples before loading them into the capillaries helps to avoid air bubbles in the capillaries.
      ***Note:*** Avoid liquid on the outside of the capillary. Do not touch the capillary in the center.
      ***Note:*** The inflection temperature at pH 8.0 was determined to be >65°C, also after storage for 31 days at 4°C (see Figure 7).

**Figure 7:**
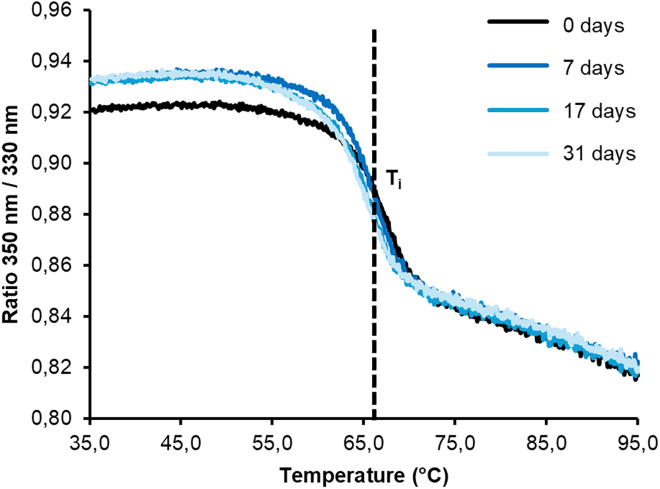
Representative unfolding curves of the purified LSAM domain The inflection temperature at pH 8.0 was determined to be >65°C, also after storage for 31 days at 4°C.
15. Confirming Protein Identity by Western blotting.
  ***Note:*** This section describes the analysis of the protein identity of the LSAM domain by Western blotting. The experiment was performed by using polyclonal anti-prolegumain antiserum generated upon immunization of rabbits with recombinant human prolegumain protein (prepared in our lab). The anti-prolegumain antibodies present in the anti-serum recognize both the catalytic domain and the LSAM domain within prolegumain (Figure 8). This analysis contains three sequential steps: Protein transfer, antibody binding, and detection.
  a. Protein transfer.
    i. Mix 2.5 μg of protein sample with an appropriate amount of 2× SDS-PAGE loading buffer containing reducing agent (DTT).
    ii. Run gel until good separation is reached.
    iii. Rinse SDS-PAGE gel in transfer buffer.
    iv. Soak SDS-PAGE gel in transfer buffer for 5 min.
    v. Soak filter paper in transfer buffer while shaking.
    vi. Rinse a PVDF membrane in 100% methanol for 15 s.
    vii. Wash the membrane with H_2_O for 2 min.
    viii. Soak the membrane in transfer buffer on the shaker.
    ix. Assemble the blot in the following order: filter paper, transfer buffer, membrane, transfer buffer, SDS-PAGE gel, transfer buffer, filter paper, transfer buffer.
    x. Close the blotting chamber.
    xi. Blot for 30 min at 15 V.
    xii. Disassemble blot and wash membrane with TBST for 5 min.
    xiii. Repeat this step 2 times.
  b. Antibody binding.
    i. Incubate the membrane with blocking solution for 1 h on a shaker.
    ii. Wash membrane with TBS for 5 min on a shaker.
    iii. Repeat this step 2 times.
    iv. Incubate membrane with primary antibody. In our case with antiprolegumain antiserum (1:500 in 0.5% w/v BSA in TBST) overnight (16 h) at 4°C on a shaker.
    v. Wash membrane with TBST for 5 min on a shaker.
    vi. Repeat this step 3 times.
    vii. Incubate membrane with secondary anti-rabbit antibody (1:30.000 in 0.5% w/v BSA in TBST) for 1 h on a shaker.
  c. Detection.
    i. Wash membrane with TBST for 5 min on a shaker.
    ii. Repeat this step 3 times.
    iii. Develop the Western Blot with ECL solution take images with the GelDoc imaging system (Bio-Rad).
      ***Note:*** A commercial anti-legumain antibody can also be used. For example, we successfully used the human Legumain/Asparaginyl Endopeptidase Antibody (catalog number: AF2199) from R&D Systems. However, in that case, care has to be taken that a suitable secondary antibody is chosen which recognizes the primary antibody.

**Figure 8:**
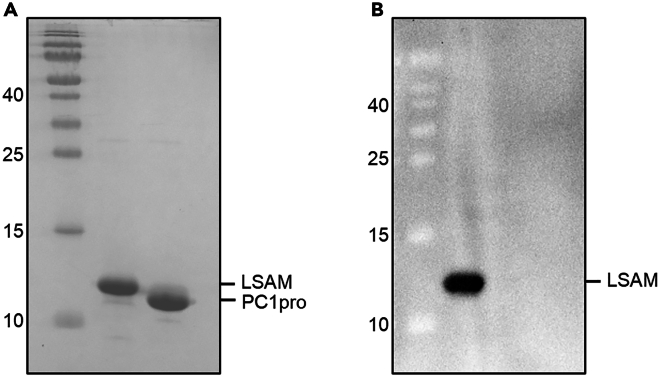
Western blot of purified LSAM domain confirms its identity The polyclonal anti-prolegumain antibodies within the anti-serum specifically recognized our recombinant LSAM domain, but not the PC1pro-fusion tag. Thereby the western blot confirms the identity of the purified LSAM domain. (A) Purified LSAM domain (lane 1) and PC1pro (lane 2) were loaded on an SDS-PAGE gel and stained using Coomassie Brilliant Blue. (B) Western blot analysis of a duplicate gel corresponding to the one shown in (A).

## Expected outcomes

The LSAM domain plays a crucial role in regulating legumain activity, which is involved in various types of cancer and neurodegenerative diseases.^14^^,^^15^^,^^16^^,^^17^^,^^18^ To date, no protocol has been available for the recombinant production of the LSAM domain in either eukaryotic or bacterial expression systems. This is due to the challenging structure of the domain, which includes two disulfide bonds. In this study, we present a step-by-step protocol for the successful expression of the LSAM domain in *E. coli*. Following this method, 2.0–2.5 mg of pure recombinant LSAM domain with intact disulfide bonds can be obtained in 7 days. Biophysical analysis, including protein purity assessment via SDS-PAGE and evaluation of proper folding through CD spectroscopy and thermal denaturation, can be completed within 1 to 2 days.

## Limitations

This protocol outlines the expression and purification of recombinant human LSAM domain in *E. coli* for biophysical and structural studies. The main step in its purification is its isolation and renaturation from non-classical inclusion bodies. The PC1-pro fusion tag proved critical to drive the formation of non-classical inclusion bodies rather than classical inclusion bodies. We anticipate this protocol to be adaptable to other LSAM domains and other disulfide-containing target proteins as well. However, as we did not systematically test it, it would certainly require optimization.

## Troubleshooting

### Problem 1

[Step 2] The expression yield is low.

### Potential solution

Check the OD600 of the *E. coli* cultures and let cells grow longer before induction until an OD of ∼10 is reached. Alternatively, a different *E. coli* strain like BL21(DE3) Rosetta may be tested as well.

### Problem 2

[Step 5] Solubilization of NCIBs did not work well and resulted in only a low amount of PC1pro-LSAM fusion protein.

### Potential solution

Increase the time for resolubilization from overnight (16 h) to e.g. 24 h. Increase concentration of Urea from 3 M to 3.5 M. Check if cell lysis was complete. If it was not, do more rounds of sonication. If the yield is still low, set up a new expression.

### Problem 3

[Step 7] Protein was not clean after Ni^2+^-purification.

### Potential solution

Include more washing steps, potentially slightly increase the concentration of imidazole in the washing buffers.

### Problem 4

[Step 8] Precipitation is observed during dialysis.

### Potential solution

Even though we did not observe protein precipitation in our dialysis reactions, it may still happen. If precipitation is observed, we recommend centrifuging the protein solution after dialysis at 17,000 g for 30 min.

### Problem 5

[Step 9] TEV-digestion is not complete.

### Potential solution

Increase incubation time and/or use a higher ratio of TEV protease to PC1pro-LSAM fusion protein. Alternatively, dialysis might be incomplete. Increase dialysis time and do another exchange of dialysis buffer.

### Problem 6

[Step 10] Separation of PC1pro-fusion tag and LSAM domain via Anion exchange chromatography does not work well.

### Potential solution

Use a different NaCl concentration to elute LSAM domain from the IEX column. To find out the correct NaCl concentration, use a linear NaCl gradient elution.

## Resource availability

### Lead contact

Further information and requests for resources and reagents should be directed to and will be fulfilled by the lead contact, Elfriede Dall (elfriede.dall@plus.ac.at).

### Technical contact

Technical information and requests should be directed to and will be fulfilled by the lead contact, Elfriede Dall (elfriede.dall@plus.ac.at).

### Materials availability

For this study the gene sequence of the LSAM domain was codon optimized for *E. coli* expression. The sequence can be found in Figure 1A. The expression plasmid is available at Addgene: 240215. Requests for material should be sent to E.D.

### Data and code availability

The data presented in this study are available on request from the corresponding author (Elfriede Dall).

## Acknowledgments

This research was funded in whole or in part by the 10.13039/501100002428Austrian Science Fund (FWF) (10.55776/Y1469 to E.D. and 10.55776/P36648 to S.O.D.).

## Author contributions

S.O.D. performed most experiments, analyzed data, and reviewed the manuscript. A.C.W. performed experiments, analyzed data, and reviewed the manuscript. H.B. analyzed data and reviewed the manuscript. E.D. supervised the study, analyzed data, and wrote the manuscript.

## Declaration of interests

The authors declare no competing interests.
